# Predictors of post-infectious chronic fatigue syndrome in adolescents

**DOI:** 10.1080/21642850.2013.869176

**Published:** 2014-01-02

**Authors:** Leonard A. Jason, Ben Z. Katz, Yukiko Shiraishi, Cynthia J. Mears, Young Im, Renee R. Taylor

**Affiliations:** ^a^ Center for Community Research, DePaul University, 990 W. Fullerton Ave, Chicago, IL60614, USA; ^b^ Division of Infectious Diseases & Department of Pediatrics, Northwestern University & Anne and Robert H. Lurie Children's Hospital of Chicago, 225 E. Chicago Ave, Chicago, IL60611, USA; ^c^ Independent Researcher, 715 Lake St., Suite 807, Oak Park, IL60301, USA; ^d^ Heartland Health Centers, 3048 N Wilton, Chicago, IL60657, USA; ^e^ Department of Occupational Therapy, University of Illinois at Chicago, 1919 W. Taylor St. (MC 811), Chicago, IL60612, USA

**Keywords:** mononucleosis, chronic fatigue syndrome, risk factors, autonomic symptoms, longitudinal

## Abstract

This study focused on identifying risk factors for adolescent post-infectious chronic fatigue syndrome (CFS), utilizing a prospective, nested case–control longitudinal design in which over 300 teenagers with infectious mononucleosis (IM) were identified through primary care sites and followed. Baseline variables that were gathered several months following IM, included autonomic symptoms, days in bed since IM, perceived stress, stressful life events, family stress, difficulty functioning and attending school, family stress, and psychiatric disorders. A number of variables were predictors of post-infectious CFS at six months; however, when autonomic symptoms were used as a control variable, only days spent in bed since mono was a significant predictor. Step-wise logistic regression findings indicated that baseline autonomic symptoms as well as days spent in bed since mono, which reflect the severity of illness, were the only significant predictors of those who met CFS criteria at six months.

Infectious mononucleosis (IM) is associated with a subsequent symptom complex, involving severe fatigue and associated physical and cognitive symptoms (Buchwald, Rea, Katon, Russo, & Ashley, 2000; Katz, Shiraishi, Mears, Binns, & Taylor, 2009; White et al., 1998). Epstein–Barr virus (EBV) is the most common cause of IM. EBV causes almost all cases of heterophile antibody positive IM, and the heterophile antibody test is positive in about 90% of young adults who develop IM (Katz, 2012).

Rates of acute, mononucleosis-like illness preceding chronic fatigue have been documented in up to 73–78% of adolescents, with almost half evidencing active mononucleosis infection at onset (Feder, Dworkin, & Orkin, 1994; Smith et al., 1991). Many adolescents with chronic fatigue syndrome (CFS) recall a sudden, infectious onset characterized by fever, pharyngitis, and lymphadenopathy (Bell, 1992; Carter, Edwards, Kronenberger, Michalczyk, & Marshall, 1995; Jordan et al., 1998; Smith et al., 1991).

Several studies have attempted to better define the relationship between EBV and CFS. White et al. (1998) assessed patients 16–65 years of age with either glandular fever (the British term for IM) or an upper respiratory tract infection (URI) for the development of fatigue and/or CFS. Nine percent of subjects with glandular fever, whether due to EBV or a different etiologic agent, were fatigued and complained of excessive sleeping at six months, compared with none in the URI group; symptoms appeared to be worse in the EBV-associated glandular fever group. Similarly, Buchwald et al. (2000) and Katz et al. (2009) found that 12% of adults and adolescents, respectively, met criteria for CFS six months following IM. Finally, Hickie et al. (2006) in the Dubbo Infection Outcomes Study showed an 11% rate of CFS six months following glandular fever (as well as two other similar, systemic infections common in Australia, Q fever and Ross River virus). In summary, about 10% of individuals do not fully recover from IM and meet the criteria for CFS six months following IM (Buchwald et al., 2000; Hickie et al., 2006; Katz et al., 2009; White et al., 1998).

Stressful life events have been implicated as an adult risk factor for CFS following viral infection (Hatcher & House, 2003; Ray, Jefferies, & Weir, 1995; Salit, 1997). For example, Theorell, Blomkvist, Lindh, and Evengard (1999) measured the relationship between CFS onset, stressful life events, and infections in 46 patients with CFS and matched controls. In comparison to controls, individuals with CFS demonstrated a greater prevalence of negative life events three months prior to CFS onset. In a pediatric sample, Pipe and Wait (1995) found that significant life events have been reported in some children prior to the development of CFS. These findings overall suggest that stressful life events may play a role in post-infectious CFS, yet they are not consistently observed (Friedberg & Jason, 1998; White et al., 1998, 2001).

Some have suggested that CFS might be a physical manifestation of family dysfunction, as CFS symptoms may be utilized for primary or secondary gain, to cope with developmental issues or change, or to deal with family problems (Barsky & Borus, 1999). However, Pelcovitz et al. (1995) found no differences between families of adolescents with CFS and families of adolescents with cancer and control families assessed on family functioning measures and marital problems indices.

Adolescents with CFS typically experience severe exhaustion, cognitive difficulties, significant educational and vocational losses, and marked disruption of social activities and relationships (Crawley, Emond, & Sterne, 2011; Kennedy, Underwood, & Belch, 2010; Nijhof et al., 2011). In addition, orthostatic intolerance has been found in most adolescents with CFS (Rowe & Calkins, 1998), and this can contribute to symptoms such as fatigue, nausea, headache, exercise intolerance, visual disturbances, sleep, and cognitive problems. Young people with CFS often have marked functional impairment as well as educational disruption (Marshall, Gesser, Yamanishi, & Starr, 1991). Dowsett and Colby (1997) identified CFS as the most common cause of prolonged medical leave from school among adolescents in the UK. One-third of adolescents with CFS report severe restrictions of all activities and marked drops in school performance; some miss up to 80 days in a six-month period (Smith et al., 1991). Moreover, Carter et al. (1995) found that 55% reported a decline in academic performance since illness onset, and 80% indicated major reduction in extracurricular activities. CFS persisting into young adulthood leads to associated social, academic, and occupational morbidity (Bell, Jordan, & Robinson, 2001; Carter & Stockhammer, 2003). Walford, Nelson, and McCluskey (1993) also found that the CFS group had significant social and academic impairment.

There might be a relationship between the severity of the inciting episode of IM and the subsequent probability of CFS. Chretien, Esswein, Holland, and McCauley (1977) showed that gastrointestinal symptoms such as anorexia, nausea or vomiting, and palatal petechiae correlated with prolonged recovery from IM. Macsween et al. (2010) found a statistically significant longer duration of fatigue following IM in females who could not walk 100 meters at the time their acute illness was most severe. Huang, Katz, Mears, Kielhofner, and Taylor (2010) found in a prospective study of teenagers with IM, that at baseline, there was a significant difference between the CFS and controls in autonomic symptomatology and fatigue levels (Huang et al., 2010), but they did not examine other baseline variables reported in the literature such as psychiatric disorders, life stressors, or school functioning, nor did it attempt to predict which young people might go on to develop CFS based on baseline variables.

Although a series of well-designed prospective studies have identified a post-infectious CFS subgroup six months following mononucleosis (Buchwald et al., 2000; Katz et al., 2009; Katz, Stewart, Shiraishi, Mears, & Taylor, 2011; White et al., 1998), there is a need for studies to evaluate predictors of the course of post-infectious CFS in that population. This study focused on a number of risk factors assessed during the first few months following IM, aside from autonomic symptoms, including perceived stress, stressful life events, family stress, academic and school function, and the presence of psychiatric disorders in the same cohort reported by Katz et al (2011). We assessed in young people with IM, the relationship between a number of baseline measures (i.e. severity of IM, perceived stress, stressful life events, family stress, academic and school function, and the presence of psychiatric disorders) and progression to CFS. We hypothesized that severity of IM illness would be the primary predictor for determining the likelihood of developing CFS.

## Methods

### Participants

In Stage 1, 301 adolescents aged 12–18 were recruited from a wide base of clinical care sources, including school-based health clinics (middle school, high school, and college/university) within the Chicago Metropolitan Area and surrounding counties (for more details of this cohort, see Katz et al., 2009, 2011). These included three large-scale primary care sources: (1) Children's Memorial (currently the Ann and Robert H. Lurie Children's) Hospital's Primary Practice Research Group, (2) Advocate Health Care, and (3) University of Illinois Family Practice and Pediatric Primary Care Service. A Recruitment Coordinator served as the primary care practice and school-based health clinic recruiter and liaison.

In the next stage, an initial baseline home-visit occurred, which included a blood draw, psychiatric interview, and an interview about symptoms and psychosocial functioning shortly after the time of infection. Baseline was a median of two months of the diagnosis of IM. Participants or their guardians choosing to respond in Spanish had the option of doing so, and all consent/assent forms and measures not already translated were translated into Spanish and back-translated into English to verify accuracy. Next, a follow-up telephone screening interview occurred six months post-infection to assess for self-reported symptoms of CFS. A complete medical and psychiatric work-up occurred for participants from the IM group that screened positive for self-reported CFS symptoms based on the six-month telephone interview and for screened-negative controls with efforts to match to subjects diagnosed with CFS by age, gender, and socioeconomic status. A larger number of controls than index cases were recruited. An independent team of physicians blind to condition reviewed each chart and reached diagnostic consensus regarding the presence or absence of CFS. The Jason et al. (2006) revision of the Fukuda (1994) criteria was used to diagnose CFS. When a well-recognized underlying condition, such as primary depression, could explain the subject's symptoms, s/he was classified as having “CFS-explained”.

## Measures

*Autonomic Symptoms Checklist – Patient Version* (ASC) was adapted from the Autonomic Symptom Profile (Suarez et al., 1999), and has been validated for CFS (Newton et al., 2007) and has been used down to age 12 (Biegstraaten, van Schaik, Wieling, Wijburg, & Hollak, 2010). Scoring was decided a priori; items were graded from 1 to 7 and then weighted from 1 to 4. The ASC was selected as a measure of the severity of illness, and it measures different types of autonomic symptoms, which can include problems with the regulation of heart rate, blood pressure, body temperature, perspiration, and bowel and bladder functions, and experiencing fatigue, lightheadedness, feeling faint or passing out, and/or weakness. This measure provides an overall score of deficits in the autonomic domain.

*The Perceived Stress Scale* (PSS) is a 4-item revised version of a longer 14-item measure of global perceived stress over the previous month (Cohen, Kamarck, & Mermelstein, 1983). The authors reported a coefficient alpha reliability of .72 for the four-item short version. The PSS-4 was used in this study. The scale ranges from 0 to 4 (0 = never; 1 = almost never; 2 = sometimes; 3 = fairly often; 4 = very often). The total stress score ranges from 0 to 16, with higher scores measuring more stress.

The *Life Events Questionnaire for Adolescents* is a questionnaire that assesses the occurrence of 13 stressful events (e.g. a close relative suffered a serious illness, your parents divorced or separated, etc.) over the past year. It was developed to examine the link between stressful life events and adolescent adjustment (Masten, Neemann, & Andenas, 1994). There are three dimensions of interest: discreteness, desirability, and independence (1994). Test–retest correlations over the course of 12 months were moderately high (Brady & Matthews, 2002). The items are summed up to provide a total score.

The checklist of infectious symptoms is a self-report measure that has been used in a large-scale adult study of CFS following IM (Buchwald et al., 2000). It is a self-report measure of the presence and severity of IM symptoms. Questions regarding functioning included “Since mono, how many days have you spent in bed?” Family Stress questions involved functioning and school-related issues during the baseline home evaluation (e.g. “Any family stress around or prior to mono onset?”) A psychiatric interview [the Structured Clinical Interview for the Diagnostic and Statistical Manual of Mental Disorders – Fourth Edition (DSM-IV) (K-SCID)] was administered during a separate session within one to two weeks of the initial visit after the diagnosis of IM was confirmed. The K-SCID is a widely used semi-structured psychiatric interview specifically designed for research with children and adolescents (Matzner, Silva, Silvan, & Chowdhury, 1997). This semi-structured clinical interview has been successfully used to assess psychiatric disorders in pediatric samples of people with chronic fatigue and CFS (Jordan et al., 2000). Diagnoses are based on adolescent self-report and any relevant contributing information about the adolescent from the parent or caretaker. Diagnoses emerging from the K-SCID conform to DSM-IV diagnostic criteria for Axis I and include: disruptive behavior disorders, mood and psychotic disorders, anxiety disorders, alcohol and substance abuse disorder, and adjustment disorder. The K-SCID has good reliability and validity and excellent inter-rater reliability (e.g. 0.84 for the disruptive behavior module and 1.0 for the attention deficit hyperactivity module) (Matzner et al., 1997).

### Statistical analysis

**Thirteen simple logistic regressions were conducted. Because there were no sociodemographic differences between groups, no control variables were entered into this equation. Thirteen separate analyses were run in order to see the total effects of each predictor. Additionally, these predictors were analyzed a second time, while statistically controlling for autonomic symptoms as measured by the ASC, so as to allow for examination of the role of autonomic symptoms which is a measure of baseline illness severity. The number of predictors was too large for our sample size to be entered simultaneously in an equation, and a high degree of multicolinearity prohibited finding unique effects. We also conducted a step-wise logistic regression using likelihood-ratio-based forward selection. For all analyses, we compared the 39 index cases to the 50 controls.

## Results

### Study cohort

**Figure 1.**
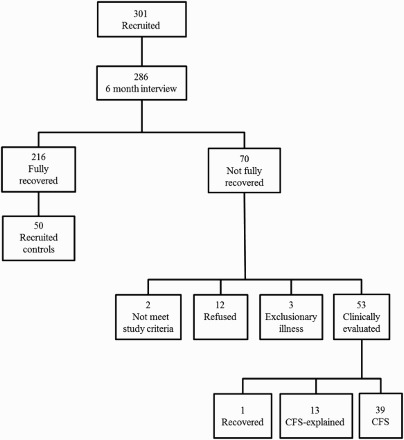
Flow sheet of youth with IM.

Figure 1 is a flow diagram for the study. Of the 301 adolescents, 286 (95%) went through the telephone screening interview six months after their IM diagnosis, and 70 (24%) were screened as not fully recovered. Of these 70 adolescents, 53 (76%) had a clinical evaluation; the other 17 adolescents refused, had exclusionary diagnoses or failed to meet criteria. There was no significant difference in sex, family socioeconomic status, or subject age between the group that completed the 6-month evaluation (*N* = 53), the group (*N* = 12) that refused or the group (*N* = 5) that was excluded. Thirty-nine out of 53 clinically evaluated adolescents who did not fully recover were diagnosed with CFS. Compared with the other enrolled subjects in the cohort, 35 of the 39 subjects with CFS at 6 months were female (90%, versus 68%, *p* =  0.01 by Fisher's exact test). There was no difference in ethnic group or socioeconomic status between the entire cohort and the subjects who went on to develop CFS. There was no difference in family socioeconomic status or subject age between the group diagnosed with CFS (*N* = 39) and the fully recovered control group (*N* = 50) (Table 1). Only those participants that rated themselves as fully recovered were considered eligible for selection as a control (for more details, see Katz et al., 2009, 2011). As our data only included youth with IM, we were not able to recruit a control group who had not had IM.

**Table 1.** Sociodemographic characteristics of the CFS and control groups.

	CFS (*N* = 39)	Control (*N *= 50)
Sociodemographic variables	*M* (SD)	*M* (SD)
*Age*	16.08 (*1.40*)	16.10 (*1.50*)
	*N (%)*	*N (%)*
*Gender*		
Male	4 (*10.3%*)	13 (*26.0%*)
Female	35 (*89.7%*)	37 (*74.0%*)
*Ethnic group*		
Caucasian	34 (*87.2%*)	47 (*94.0%*)
African-American	3 (*7.7%*)	1 (*2.0%*)
Latino	0 (*0.0%*)	1 (*2.0%*)
Other	2 (*5.1%*)	1 (*2.0%*)
*SES*^a^		
Unskilled laborers	1 (*2.6%*)	1 (*2.0%*)
Machine operators	9 (*23.1%*)	7 (*14.0%*)
Skilled craftsmen	13 (*33.3%*)	20 (*40.0%*)
Minor professional	9 (*23.1%*)	12 (*24.0%*)
Professional	5 (*12.8%*)	6 (*12.0%*)

^a^Based on the parents' occupation.

As seen from Table 2, a number of baseline variables were predictors of the diagnosis of post-infectious CFS at six months. However, when the ASC was used as a control variable, only days spent in bed since IM was a significant predictor. Finally, as seen from Table 3, when using step-wise logistic regression, the only significant predictors were ASC and how many days were spent in bed since IM, indicating the importance of baseline severity variables. The variable “Any family stress around or prior to IM onset” was not found to be significant.

**Table 2.** Simple logistic regression results predicting diagnosis.

	Without controlling for ASC					Controlling for ASC				
Predictor baseline variables	*b*	SE	Wald *χ*^2^	*p*-Value	OR	*b*	SE	Wald *χ*^2^	*p*-Value	OR
*Autonomic symptoms*										
ASC	.14	.03	22.23	.00	1.15					
*Perceived stress*										
Perceived stress score	.10	.03	9.81	.00	1.10	.06	.04	2.35	.12	1.06
*Life events*										
Life events score	.60	.17	13.14	.00	1.83	.34	.20	2.89	.09	1.41
*Family stress*										
Any family stress around or prior to mono onset?	.00	.00	2.79	.09	1.00	.00	.00	2.08	.15	1.00
If yes, is it still continuing?	.00	.00	1.68	.19	1.00	.00	.00	.34	.56	1.00
Since mono, has there been stress in your family?	.00	.00	2.73	.10	1.00	.00	.00	2.06	.15	1.00
If yes, is it still continuing?	.00	.00	3.18	.07	1.00	.00	.00	.51	.47	1.00
*Difficulty functioning/attending school*										
Since mono, how many days have you spent in bed?	.08	.03	5.98	.01	1.08	.10	.05	4.85	.03	1.11
Since mono, how many days of school have you missed?	.09	.04	5.89	.01	1.09	.08	.05	2.68	.10	1.09
Hard time attending school regularly?	.00	.00	.03	.86	1.00	.00	.00	2.04	.15	1.00
Difficulties with concentrating, learning or remembering?	.63	.43	2.07	.15	1.87	.48	.54	.80	.37	1.62
*Psychiatric diagnosis*										
Did participant receive at least one current diagnosis	1.39	.45	9.28	.00	4.00	.95	.55	2.98	.08	2.60
Total number of current diagnoses received	.68	.30	5.13	.02	1.97	.34	.34	.97	.32	1.41

**Table 3.** Likelihood-ratio-based forward selection step-wise logistic regression predicting diagnosis.

Step	Variables	*b*	SE	Wald *χ*^2^	*p*-Value	OR
*Step 1*						
	ASC	.18	.04	18.93	.00	1.20
*Step 2*						
	ASC	.21	.05	17.58	.00	1.24
	Any family stress around or prior to mono onset?	.00	.00	0.05	.82	1.00
*Step 3*						
	ASC	.25	.07	14.56	.00	1.29
	Any family stress around or prior to mono onset?	.00	.00	0.11	.74	1.00
	Since mono, how many days have you spent in bed	.14	.07	3.89	.05	1.15

Note: −2 Log likelihood for step 1 was 58.50, *χ* ^2^(1) = 34.85, *p* = .00, for step 2, Δ*χ* ^2^(1) = 10.96, *p* = .00, for step 3, Δχ^2^(1) = 6.73, *p* = .01.

## Discussion

IM appears to be a predisposing factor for some individuals who develop CFS, especially adolescents (Feder et al., 1994; Smith et al., 1991). Many candidate risk factors have been proposed to explain this phenomenon, but almost all lack prospective data from before IM or CFS. According to this study, significant baseline predictors in the step-wise logistic regression included autonomic symptoms and days spent in bed since the onset of IM. This suggests that indices of illness severity are the best predictors for adolescents destined to develop CFS following IM. It is reasonable to conclude from our study that during the first few months following IM, young people who have more limitations and are more impaired, are subsequently more likely to develop CFS. Our findings are thus comparable to those of Hickie et al. (2006), who followed patients with mononucleosis (glandular fever), Q fever, and Ross River virus who later met criteria for CFS. Development of CFS in their cohort was predicted largely by the severity of the acute illness rather than by demographic, psychological, or microbiological factors.

Psychological distress has been found to play a significant role in relation to the course of oral and genital herpes virus reactivation, exacerbation of HIV and the development of IM (Carver, Connallon, Flanigan, & Crossley-Miller, 1994; Cohen & Williamson, 1991; Glaser et al., 1991; Imboden, Canter, & Cluff, 1961; Kasl, Evans, & Niederman, 1979; Perry, Fishman, Jacobsberg, & Frances, 1992). However, Hickie, Koschera, Hadzi-Pavlovic, Bennett, and Lloyd (1999) found that chronic fatigue is a persistent diagnosis over time and that longitudinal patterns of comorbidity of fatigue with psychological distress did not suggest a causal relationship or common vulnerability factor. This study also did not find that psychiatric disorders assessed a few months after developing IM were associated with the development of CFS, after controlling for ACS.

Several studies have identified family stress as a precursor to CFS (Carter et al., 1999; Van Middendorp, Geenen, Kuis, Heijnen, & Sinnema, 2001). The studies by van Middendorp and Carter were of children referred to psychologists, in whom one would anticipate a higher rate of behavioral factors. In these studies, along with a potential for referral bias, these adolescents most likely are not representative of the CFS population as a whole. Taylor, Jason, and Jahn (2001) found prevalence rates of sexual and physical abuse among individuals with CFS comparable to those found in individuals with other conditions involving chronic fatigue. Our study also did not show a relationship between familial stress and the development of post-infectious CFS.

Brown, Bell, Jason, Christos, and Bell (2012) examined long-term outcomes of 25 people who were diagnosed with CFS while they were adolescents, approximately 25 years ago. Of the 25 participants, only 5 self-reported maintaining that diagnosis, while 20 reported remission. In spite of their self-reported remission, however, those 20 participants showed significantly more impairment compared with controls, demonstrating that, while adolescents diagnosed with CFS may show improvement over time, they still suffer some level of impairment and may not return to their premorbid level of functioning. Clearly, given the long-term effects of CFS, it is critical to better understand potential risk factors associated with this illness.

It is important to note that the baseline visit occurred within a median of two months of the diagnosis of IM, but the diagnosis of mono itself would have taken some additional time. A reasonable assumption about IM in adolescents is that symptoms are present for up to a month before diagnosis, making the time for administration of the questionnaires up to three months after the onset of illness. Therefore, some of the stressful life events recorded might have occurred in the three months after the onset of IM and might, therefore, have been influenced by the illness severity rather than by prior family stress. Questions on the PSS begin with the wording: “In the last month, how often have you … ” Due to the timing of enrollment, the perceived stress may also be more likely to reflect the stress caused by the illness rather than perceived stress when the individual was healthy. Thus, our study cannot address the issue of whether pre-IM stress is a risk factor for developing CFS following IM in adolescents. While it is true that the Life Events Questionnaire for Adolescents asked about life events in the preceding year, we were not able to specifically tease out those events that occurred prior to the onset of IM. It is also important to differentiate difficulty in school functioning (which infers a behavioral problem) from illness severity as risk factors for the development of CFS following IM, which this study was also unable to accomplish.

This study has several other limitations, including modest sample size and data only at a six-month assessment following IM. In addition, the sample was relatively homogenous in terms of gender and ethnic group. There is a need for more long-term studies with larger community-based samples in order to better identify the predisposing medical and psychological risk factors involved in the development of pediatric CFS. Future studies might examine biological data on symptom severity at onset of IM (such as thromobocytopenia or anemia) in those who develop CFS versus those that do not following IM.

The relationship between IM and CFS needs to be fully understood; not only for comprehending the relationship between the two illnesses, but also for healthcare-provider guidance to adolescents and their parents. The prevention of the progression from IM to CFS not only saves the patient from the potential of lifelong disability, financial dependency, and the potential for ensuing depression, but may save the family from life-altering care-giving and financial responsibilities; the stresses of which alter the family dynamics so drastically and detrimentally that the family unit itself may not survive.
